# A Naturally Occurring Null Variant of the NMDA Type Glutamate Receptor NR3B Subunit Is a Risk Factor of Schizophrenia

**DOI:** 10.1371/journal.pone.0116319

**Published:** 2015-03-13

**Authors:** Hitomi Matsuno, Kazutaka Ohi, Ryota Hashimoto, Hidenaga Yamamori, Yuka Yasuda, Michiko Fujimoto, Satomi Yano-Umeda, Takeo Saneyoshi, Masatoshi Takeda, Yasunori Hayashi

**Affiliations:** 1 Brain Science Institute, RIKEN, Wako, Saitama, 351-0198, Japan; 2 Department of Psychiatry, Osaka University Graduate School of Medicine, Osaka, 565-0871, Japan; 3 Molecular Research Center for Children's Mental Development, United Graduate School of Child Development, Osaka University, Osaka, 565-0871, Japan; 4 Department of Molecular Neuropsychiatry, Osaka University Graduate School of Medicine, Osaka, 565-0871, Japan; 5 Saitama University Brain Science Institute, Saitama University, Saitama, 338-8570, Japan

## Abstract

Hypofunction of the *N*-methyl-D-aspartate type glutamate receptor (NMDAR) has been implicated in the pathogenesis of schizophrenia. Here, we investigated the significance of a common human genetic variation of the NMDAR NR3B subunit that inserts 4 bases within the coding region (insCGTT) in the pathogenesis of schizophrenia. The cDNA carrying this polymorphism generates a truncated protein, which is electrophysiologically non-functional in heterologous expression systems. Among 586 schizophrenia patients and 754 healthy controls, insCGTT was significantly overrepresented in patients compared to controls (odds ratio = 1.37, *p* = 0.035). Among 121 schizophrenia patients and 372 healthy controls, genetic analyses of normal individuals revealed that those carrying insCGTT have a predisposition to schizotypal personality traits (*F_1,356_* = 4.69, *p* = 0.031). Furthermore, pre-pulse inhibition, a neurobiological trait disturbed in patients with schizophrenia, was significantly impaired in patients carrying insCGTT compared with those with the major allele (*F_1,116_* = 5.72, *p* = 0.018, *F_1,238_* = 4.46, *p* = 0.036, respectively). These results indicate that a naturally occurring null variant in NR3B could be a risk factor of schizophrenia.

## Introduction

Schizophrenia is a common psychiatric disease of juvenile to adult onset characterized by positive symptoms such as delusions, hallucinations, thought disorder and disorganized behavior as well as negative symptoms such as blunted emotional response, restriction in fluency and productivity of thought and speech, and impairment in initiation of goal-directed behavior. Its lifetime occurrence is 3.9% [1], affecting 240 million individuals worldwide as estimated by the World Health Organization. While there are cases where drugs and psychological treatments are effective, the remaining cases are refractory to any form of treatment and its chronic nature requires prolonged care. It leads to major family and social burden and therefore, its underlying mechanism of pathogenesis and effective treatments have been actively sought.

The hypofunction of glutamatergic transmission has been implicated in schizophrenia [2]. The first evidence supporting this idea came from a finding that phencyclidine and ketamine, two dissociative anesthetics that induce schizophrenia symptoms in individuals without past history, turned out to be channel blockers of NMDAR [3,4]. Consistently, animal models of NMDAR hypofunction by genetic down-regulation of NMDAR expression shows traits resembling schizophrenia [5,6]. Autopsy studies also revealed reduced expression of NMDAR in patients’ brain compared with age-matched controls [7]. These observations lead to an attempt to compensate the reduced NMDAR with positive modulators to treat schizophrenia [8].

NMDAR is composed of a tetrameric combination of NR1 (GluN1), NR2A-D (GluN2A-D) and/or NR3A-B (GluN3A-B) subunits [9]. NR1 is an obligatory subunit required for all NMDAR channels, while NR2 and NR3 add functional diversity observed among different neuronal cell types and developmentally regulated. The majority of neuronal NMDAR are composed of two NR1 and two NR2 while those containing NR3 subunits are limited to particular cell types and ontogenic stages. When NR3 forms a heterooligomer with NR1 and NR2, it works in a dominant-negative fashion to reduce Ca^2+^-permeability and overall current [10–12]. In contrast, when NR3 forms a heterooligomer only with NR1, it forms an excitatory glycinergic receptor [13], though the presence of synapses that contain such receptors have not been fully demonstrated in native tissue.

Genetic studies on schizophrenia implicate a strong genetic component in the pathogenesis of schizophrenia. Monozygotic twins show ∼50% concordance, while dizygotic twins show ∼17% [14]. Because of the evidence outlined above implicating the hypofunction of NMDAR in the pathogenesis of schizophrenia, association studies on NMDAR subunit genes with schizophrenia traits have been conducted. Such studies reported that polymorphisms found in both NR1, NR2, and NR3A are indeed risk factors of schizophrenia [15–21].

**Fig 1:**
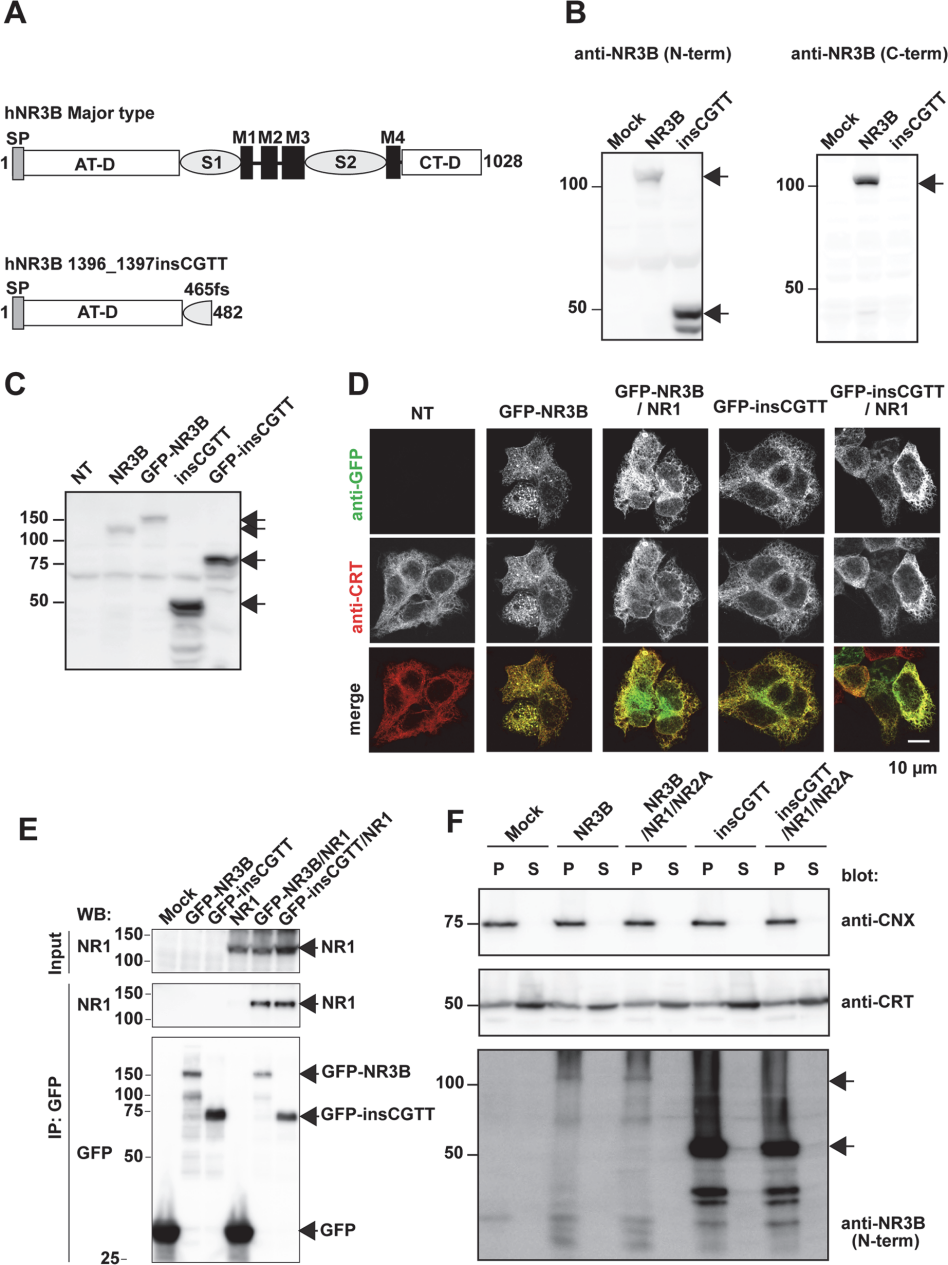
NR3B with insCGTT type generates a truncated protein that accumulates intracellularly. **A**, Schematic drawing of NR3B illustrating the position of insCGTT type. SP: signal peptide, AT-D, amino-terminal domain; S1 and S2, S1 and S2 lobes of ligand binding domain; M1-4, transmembrane and membrane associated regions; CT-D, carboxyl-terminal domain. **B**, Western blot of total lysate of HEK293T cells expressing either NR3B major type (NR3B) or insCGTT type. **C**, Western blot of lysate of HEK293T cells expressing major type NR3B, GFP-tagged major type NR3B, insCGTT type, and GFP-tagged insCGTT type, blotted with anti-NR3B N-terminus antibody. Both GFP tagged major type and insCGTT type constructs generated bands that were detected at ∼27 kD more than the respective untagged proteins. **D**, Double immunostaining of HEK293T cells expressing GFP-NR3B with GFP antibody (green) and an antibody against ER marker calreticulin (CRT, red). NT: no transfected cells. **E**, Co-immunoprecipitation of NR3B and NR1 from HEK293T cells. GFP-tagged NR3B major type or insCGTT was coexpressed with NR1 and immunoprecipitated by anti-GFP antibody. The immunoprecipitate were blotted with anti-NR1 (middle), and anti-GFP (bottom) antibodies. Immunoblot of NR1 in total cell lysate is also shown (top). The results demonstrate that both NR3B major type and insCGTT types interact with NR1 to a similar degree. **F**, Extraction of ER and Golgi luminal proteins using sodium carbonate buffer. Crude membrane fractions were treated with sodium carbonate buffer and centrifuged. Western blots were performed using the pelleted membrane fraction (P) and supernatant (S) with indicated antibodies. The detection of CTR, an intraluminal protein, but not calnexin (CNX), a representative integral luminal membrane protein, in the supernatant, indicates the successful extraction of luminal proteins. Under these conditions, both major type and insCGTT type remained in the membrane fraction and were not detect in the supernatant.

NR3B is abundantly expressed in α-motoneurons but also in other areas such as forebrain (including hippocampus, cerebral cortex, caudoputamen, and nucleus accumbens), and cerebellum, at lower levels [12,22–26]. We previously found that the gene encoding NR3B, *GRIN3B* is highly heterogeneous in humans compared with other taxa [27]. Among various genetic variants in *GRIN3B*, we found a frame-shift variant, c.1396_1397insCGTT (rs10666583, insCGTT), which inserts four bases into the middle of the coding region and leads to the premature termination of the open reading frame (Fig. 1A). This leaves the extracellular amino-terminal domain (AT-D), a region with homology with bacterial soluble periplasmic binding proteins [9]. About 10% of the normal European descendants in the United States of America have the homozygous insCGTT allele [27]. In Japanese and other East Asians, the occurrence is lower (allele frequency of insCGTT being 0.082 among Japanese and a calculated homozygous insCGTT genotype of 0.67%) [27]. In mouse, the knockout of this gene results in changes in home cage activity, anxiety-related behavior and social interaction, in addition to motor-related phenotypes, such as a moderate but significant impairment in motor learning or coordination [28]. Therefore, it is especially intriguing to understand the psychiatric and psychological consequences of the naturally occurring frame-shift variant of NR3B in humans.

Here we tested the impact of the insCGTT variation of NR3B in human psychiatric and psychological traits in schizophrenia patients and healthy individuals in Japan. We first confirmed that the insCGTT variation leads to a functionally null protein in a heterologous expression system. Then we found that schizophrenia patients have higher allele frequency of insCGTT than healthy individuals. Among healthy individuals, those with the insCGTT allele showed stronger schizophrenia traits in the Schizotypal Personality Questionnaire (SPQ) and the Wisconsin Card Sorting Test (WCST) than those with the major allele. Finally, patients carrying the insCGTT allele have a significant impairment in the pre-pulse-inhibition (PPI) test. From these observations, we conclude that the insCGTT variation of *GRIN3B* results in a functionally null NR3B protein, which constitutes a risk factor for schizophrenia.

## Materials and Methods

### Ethics statement

This study was performed in accordance with the World Medical Association’s Declaration of Helsinki and approved by the Osaka University Research Ethics Committee (genetic studies, Permit Number: 473) and RIKEN (*in vitro* studies, Permit Number: 2012-015(19)). Written informed consent was obtained from all subjects after the procedures had been fully explained. For subjects younger than 16 years old, patients with mental retardation or other medical conditions that could possibly impair understanding of the content of the consent form, and those currently in involuntary hospitalization, the consent was obtained from a family member or other legal guardian. The attending doctor was also consulted on whether the patients’ status was appropriate to participate in this study.

### cDNA

The NR3B cDNA isolated from human retinoblastoma cell line, Y79, was obtained from RIKEN Bioresource Center (clone ID RBd38E01). Compared with the NR3B cDNA sequence in Genbank (accession number NM_138690), our cDNA has both synonymous and non-synonymous polymorphisms, some of which had been already found in our past sequencing studies[27] or in the Single Nucleotide Polymorphism Database, while others were unreported (S1 Table). The c.1396_1397insCGTT mutation (dbSNP accession number rs10666583) was introduced using a standard PCR mediated method. The cDNAs were transferred to pDEST12.2 vector and used for expression in HEK293T cells. Transfection was done using XtremeGENE 9 (Roche Applied Science). Human NR1–3a (Genbank accession NM_007327.1) and NR2A (Genbank accession NM_000833.2) were obtained from NITE Biological Resource Center of Japan (NBRC) and expressed in pDEST12.2 vector. To test the cellular protein distribution and electrophysiological properties of NR3B, we used NR1–3a, a splice variant of NR1 because it was reported to show larger current response than other variants in HE293T cells [29]. The transfection ratio of NR1:NR3B (either major type or insCGTT) or NR1:NR2A:NR3B was always 1:1 or 1:1:1 according to weight of vector DNA [30]. This gives the same cDNA copy number for major and insCGTT types, as they are the same length.

GluR1 tagged N-terminally with GFP was reported previously [31]. To construct the NR3B N-terminally tagged with GFP in mature proteins, the GluR1 sequence following the signal peptide in GFP-GluR1 was replaced with a corresponding region of NR3B. To construct the GluR1 C-terminally tagged with GFP, a full-length cDNA encoding rat GluR1 was inserted in pEGFP-N1 expression vector in-frame. These cDNAs were expressed in pCAGGS mammalian expression vector [32].

### Immunoblotting and immunostaining

Antibodies were obtained from the following sources and used at the dilution indicated below. Rabbit anti-NR3B N-terminus antibody (H-230, 1:250, Santa Cruz), rabbit anti-NR3B C-terminus antibody (AP12291b, 1:200, Abgent), rabbit anti-GFP (A-6455, 1:100, Invitrogen), rat anti-GFP (GF090R, 1:500, nacalai tesque), rabbit anti-GFP (598, 1:1000, MBL), rabbit anti-calreticulin (PA3-900, 1:1000, ABR), mouse anti-calnexin (610523, 1:100, BD Bioscience) and mouse anti-NR1 (05–432, 1:1000, Millipore).

HEK293T cells expressing major type NR3B or ins CGTT type were lysed in buffer containing 1% TritonX-100, 50 mM Tris HCl, 150 mM NaCl, 10 mM NaF, and 10% glycerol, protease inhibitor cocktail (nacalai tesque), pH 8.0, centrifuged at 16,000 g to remove nuclei, and the supernatant was used for western blots. For sodium carbonate extraction (Fujiki et al., 1982), the cells were washed with phosphate buffered saline (PBS), then with 100 mM NaCl. Cells were scraped into 100 mM sodium carbonate (pH 11.5), and lysed mechanically with a glass-Teflon homogenizer, followed by sonication. The cell lysates were incubated on ice for 30 min, then ultracentrifuged at 240,000 g for 1 h. The pellet and the supernatant were used for western blots. For surface biotinylation, HEK293T cells transfected with GFP-tagged NR3B major type or insCGTT in combination with NR1 were biotinylated with 0.5 mg / ml EZ-link sulfo-NHS-SS-biotin (Pierce) in PBS, pH 8.0 for 30 min at 4°C. The reaction was terminated by adding of 50 mM Tris-HCl. The cells were washed four times with washing buffer (50 mM Tris HCl, pH 7.5, 115 mM NaCl) [33,34] and lysed with RIPA buffer (50 mM Tris HCl, pH 8.0, 150 mM NaCl, 1% NP-40, 0.5% deoxycholate, 1 mM EDTA, and 0.1% SDS). The biotinylated proteins were precipitated by mixing with immobilized NeutrAvidin beads (Pierce) for 1 h at room temperature. The beads were washed with RIPA buffer four times, then eluted for 5 min at 95°C with 2x Laemmli SDS sample buffer. Both total cell lysate and biotinylated protein were used for immunoblotting.

For immunoprecipitation, the transfected cells were lysed with 1% deoxycholate lysis buffer (50 mM Tris HCl, pH 8.5, 150 mM NaCl, 1% deoxycholate, 10% glycerol, 1 mM Na_3_VO_4_, 10 mM NaF, 1 mM β-glycerophosphate). The samples were sonicated, and centrifuged at 16,000 g for 10 min at 4°C, and the soluble fraction was incubated with GFP-TrapA (ChromoTek) at 4°C for 1–2 h. The beads were washed three times with 1% deoxycholate lysis buffer, then resuspended in 2x Laemmli SDS sample buffer. The samples were heated at 95°C for 5 min and subjected to SDS-PAGE.

For surface immunostaining, the cells were washed with cold buffer containing 144 mM NaCl, 5 mM KCl, 2 mM CaCl_2_, 10 mM HEPES, and 10 mM glucose, pH 7.4 and treated with blocking solution (4% bovine serum albumin in the same buffer) for 10 min on ice. The cells were incubated with blocking solution containing rabbit anti-GFP antibody for 1 hour on ice, and fixed with 4% paraformaldehyde/ 4% sucrose in PBS on ice for 10 min. The cells were washed and further incubated with Alexa 594 conjugated anti-rabbit antibody overnight at 4°C. Images were taken with a confocal microscope (FV 1000, Olympus).

### Electrophysiology

Electrophysiological recordings from HEK293T cells were conducted as described previously [12,27]. In brief, HEK293T cells were transfected with expression vectors of human NR1 and human NR2A, along with GFP (pCAGGS-GFP) to identify the transfected cells, without or with the major type NR3B or the insCGTT type. After 1–2 days, the whole-cell current evoked by puff-applied 1 mM glutamate was recorded. The internal and external solutions were made as described in previous reports [12,27].

### Subjects

The subjects of the genetic association study were 586 patients with schizophrenia [51.7% male (number of male/female = 303/283), mean age ± standard error (SE); 45.8 ± 0.7] and 754 healthy subjects [45.2% male (341/413), mean age ± SE; 55.5 ± 0.8]. The mean age and sex ratio were significantly different between the groups (*z* = -8.66, *p*<0.001, *χ* ^*2*^ = 5.55, *p* = 0.018). The subjects were all biologically unrelated and were Japanese. The subjects were recruited from both outpatient and inpatient units at Osaka University Hospital and other psychiatric hospitals. Each patient with schizophrenia had been diagnosed by at least two trained psychiatrists by unstructured clinical interviews, according to the criteria of the *Diagnostic and Statistical Manual of Mental Disorders*, *Fourth Edition* (DSM-IV). Healthy subjects were recruited through local advertisements. Psychiatrically healthy subjects were evaluated using unstructured interviews to exclude individuals with current or past contact with psychiatric services or with experience of psychiatric medication. We did not assess the controls for their family history of mental disorders, such as schizophrenia, bipolar disorder, or major depressive disorder. The ethnicity was determined by self- report and was not confirmed by genetic analyses.

The subjects for analyses of three phenotypes related to schizophrenia consisted of 121 patients with schizophrenia [62.0% males (75/46), mean age ± SE; 36.7 ± 1.2] and 372 healthy subjects [45.7% males (170/202), mean age ± SE; 35.9 ± 0.6]. These subjects were included in the genetic association analysis. The SPQ was administered to 361 healthy subjects [45.2% male (163/198), mean age ± SE: 36.1 ± 0.6]. The PPI was administered to 121 patients with schizophrenia [62.0% males (75/46), mean age ± SE; 36.7 ± 1.2]. The WCST was administered to 243 healthy subjects [46.1% male (112/131), mean age ± SE: 34.5 ± 0.8]. The subjects included in these analyses met additional criteria. Each patient with schizophrenia had been diagnosed by at least two trained psychiatrists according to the criteria from the DSM-IV based on the Structured Clinical Interview for DSM-IV (SCID). Healthy controls were psychiatrically, medically and neurologically evaluated using the SCID-non-patient edition to exclude individuals who had received psychiatric medications or who had first- or second-degree relatives with psychiatric disorders. Additionally, subjects were excluded from these analyses if they had neurological or medical conditions that could have potentially affected their central nervous system, such as atypical headaches, head trauma with loss of consciousness, chronic lung disease, kidney disease, chronic hepatic disease, thyroid disease, active cancer, cerebrovascular disease, epilepsy, seizures, substance-related disorders or mental retardation.

### Genotyping

The insCGTT variant is located on exon 3 (between chromosomal position 1004896 and 1004897) in the *GRIN3B* on chromosome 19p13.3. Venous blood was collected from the subjects, and genomic DNA was extracted from whole blood according to standard procedures. The variant was genotyped using the Custom TaqMan 5’-exonuclease allelic discrimination assay (Applied Biosystems, Foster City, CA, USA), as previously described [35,36]. Detailed information on the PCR conditions is available upon request. No deviation from the Hardy-Weinberg equilibrium (HWE) was detected in the patients or in controls (*p*>0.10). To increase the statistical power and decrease type I errors and based on genetic association analysis, homozygotes and the heterozygotes for the minor insCGTT allele groups were combined and treated as insCGTT allele carriers. We contrasted three phenotypes between insCGTT allele carriers and individuals without insertion.

### Schizotypal personality trait assessment

A full Japanese version of the SPQ was administered to healthy subjects [37,38]. The SPQ is a 74-item self-report questionnaire with a “yes/no” response format [39]. All items answered “yes” were scored 1. The SPQ measures nine subscales of specific schizotypal features. The total SPQ score was obtained by summing the scores from all of the items. The three schizotypal trait factors, cognitive/perceptual, interpersonal and disorganization, were derived by summing the related subscale raw scores according to the three-factor model of Raine and colleagues [40].

### Startle response measurement

A computerized human startle response monitoring system (Startle Eyeblink Reflex Analysis System Map1155SYS, NIHONSANTEKU Co., Osaka, Japan) was used to measure PPI. The methods for the startle paradigm, eyeblink acquisition, scoring parameters, and the procedure are described in detail elsewhere [41–43]. According to a previous study [44], the following startle measures were calculated: (i) acoustic startle reflex; (ii) habituation of the startle response; (iii) PPI82, PPI86, PPI90: pre-pulse inhibition at pre-pulse intensities of 82 dB, 86 dB, and 90 dB sound pressure levels (SPL), respectively.

### Assessment of executive function

To assess executive function, including cognitive flexibility in response to feedback, a modified and computerized Japanese version of the Wisconsin Card Sorting Test (Keio Version) (WCST) was administered to healthy subjects [45]. The outcome measures were numbers of Categories Achieved (CA), Total Error (TE) and Perseverative Errors of Nelson (PEN) [46]. CA is the number of categories for which six consecutive correct responses are achieved (maximum CA is 8), TE is the total number of incorrect responses (maximum TE is 48), and PEN is the number of incorrect responses in the same category as the immediately preceding incorrect response (maximum PEN is 47).

### Statistical analysis

**Fig 2:**
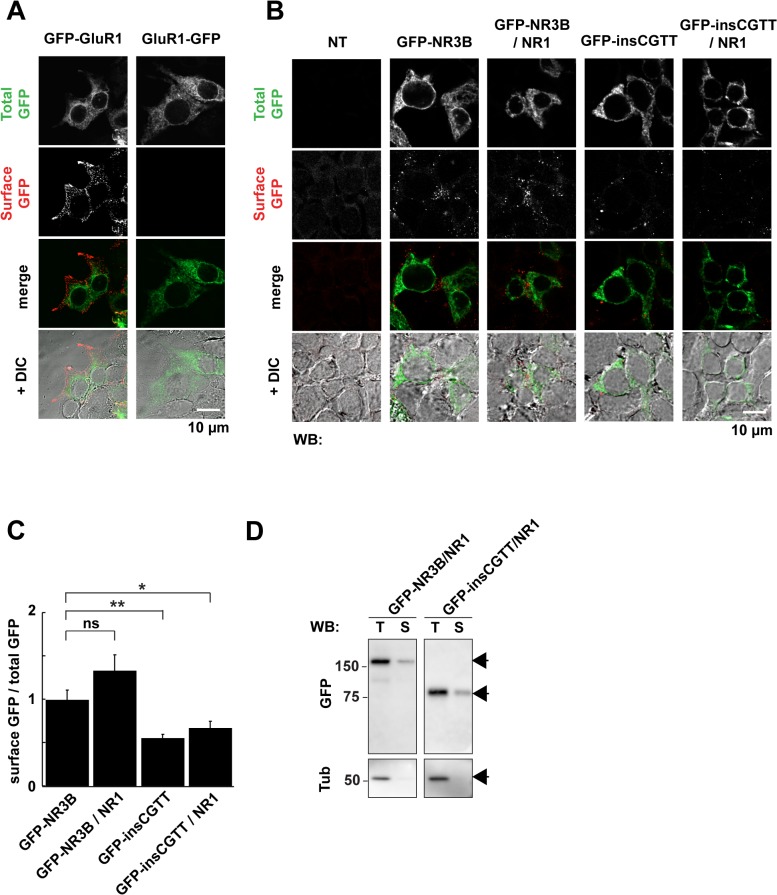
Effect of insCGTT type on distribution of NR3B in HEK293T cells. **A**, HEK293T cells transfected with GluR1 tagged with GFP at the extracellular N-terminus or intracellular C-terminus were stained with a GFP antibody under non-permeabilized conditions on ice, as verification of the specificity of cell surface receptor detection using immunostaining. **B**, HEK293T cells transfected with GFP-tagged NR3B major type or insCGTT type (green) were stained with GFP antibody under non-permeabilized condition to detect the cell surface population (surface GFP, red). **C**, Quantification of the surface levels of NR3B major type and insCGTT type with and without NR1. Because expression level of major type and insCGTT were different, the level was normalized by total GFP fluorescence in each cell. Data are expressed as a normalized value to that of major type alone. n = 55, 52, 59, and 52 for GFP-NR3B major type only, GFP-NR3B major type with NR1, GFP-NR3B insCGTT type only, and GFP-NR3B insCGTT type with NR1, respectively. *: p < 0.05; **: p < 0.01; ns: not significant. **D**, Surface biotinylation of NR3B major type and insCGTT type. GFP-tagged NR3B major type or insCGTT type was coexpressed with NR1 in HEK293T cells and the surface population was labeled by biotin. NR3B types in both total (T) and biotinylated surface population (S) were detected by anti-GFP antibody. Total fractions represent 1/8 of the starting material applied in surface fractions. α-Tubulin (Tub), an intracellular protein, was not biotinylated, confirming specificity of surface biotinylation.

**Fig 3:**
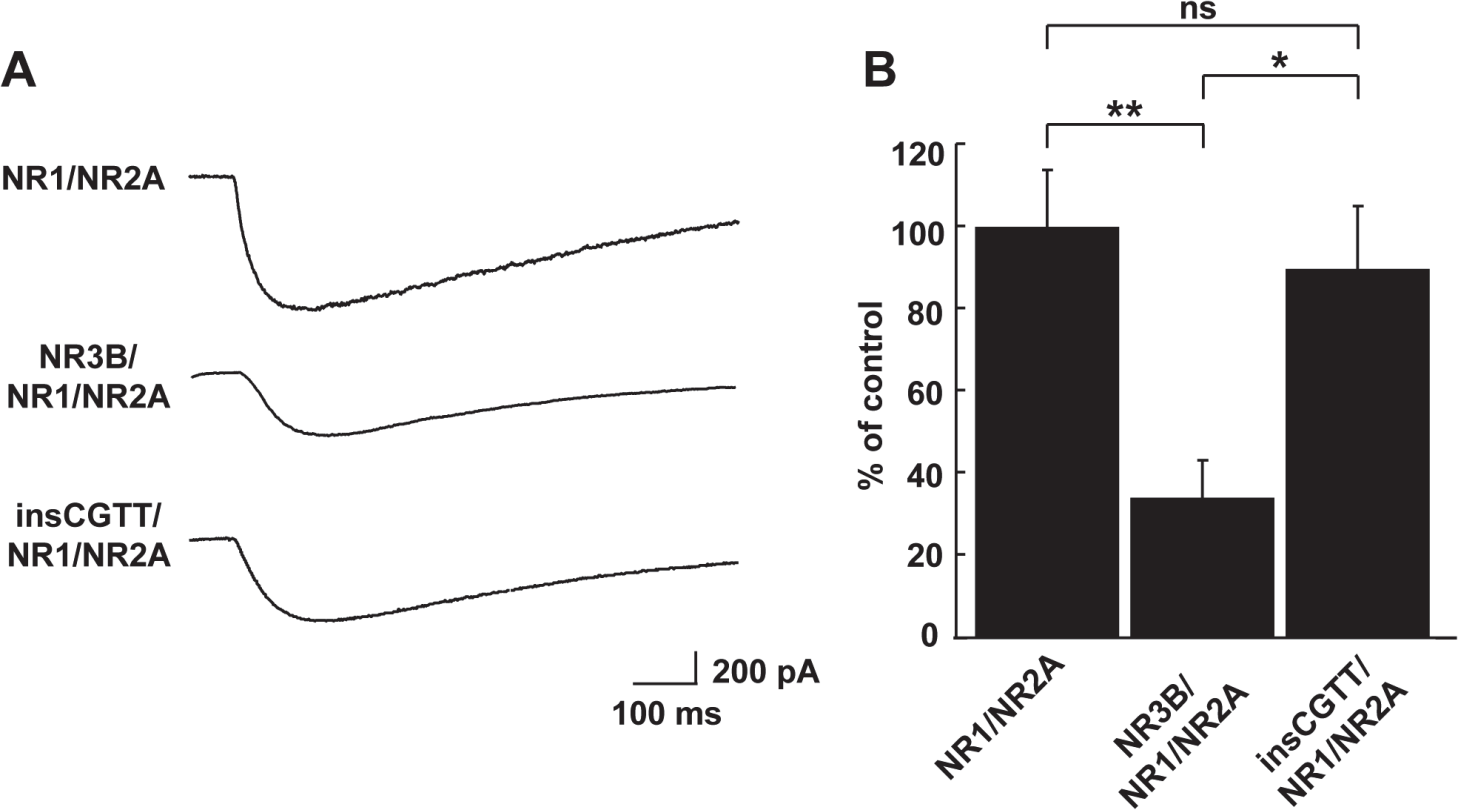
Glutamate-induced whole-cell current recorded from HEK293T cells expressing major type or insCGTT type NR3B. **A**, Sample traces of glutamate-induced whole-cell current recorded in HEK293T cells coexpressing NR1 and NR2A with or without NR3B major type or insCGTT type. The recordings were performed at -60 mV in the presence of 10 μM glycine and in the absence of Mg^2+^. **B**, The normalized current amplitude from cells without NR3B, with NR3B major type or insCGTT type. The major type NR3B suppressed the current to a statistically significant level, whereas insCGTT type did not show such suppression. The time course of current decay reflects the clearance of puffed glutamate from extracellular fluid by perfusion rather than the kinetics of channel opening and closing. n = 14, each. **: p<0.01 from control, *: p<0.05 from major type, ns: not significant.

*T*-Test and ANOVA were used in Fig. 2D and Fig. 3B, respectively. Data were represented as mean ± standard error of mean. Other statistical analyses were performed using PASW Statistics 18.0 software (SPSS Japan Inc., Tokyo, Japan). Based on the assumption that most of the clinical demographic variables, such as age and education years, were not fitted to a normality distribution with the Kolmogorov-Smirnov test (*p*<0.05), differences in clinical characteristics between patients and controls were analyzed using the non-parametric Mann-Whitney *U*-test for continuous variables, such as age and years of education, and *χ* ^*2*^ tests for categorical variables, such as sex. The presence of HWE was examined using the *χ* ^*2*^ test for goodness-of-fit via SNPAlyze V5.1.1 Pro software (DYNACOM, Yokohama, Japan). The allelic and genotypic distributions of the insCGTT variant between patients and controls were analyzed using Fisher’s exact tests with the SNPAlyze software. The effects of the insCGTT variant on schizotypal personality traits, PPI and executive function were analyzed by a one-way analysis of covariance (ANCOVA). To control confounding factors, age, sex and education years were used as covariates. As education years were highly correlated with estimated premorbid IQ, we included only education years of these two confounding factors as the covariates. All *p* values are two tailed, and statistical significance was defined as *p*<0.05.

## Results

### Subcellular distribution of NR3B insCGTT type protein in heterologous cells

In order to characterize the NR3B protein derived from the insCGTT type allele, we expressed human NR3B cDNA without insCGTT (major type) and with insCGTT in HEK293T cells. Western blots were performed with two different antibodies, one recognizing the N-terminal extracellular domain and one recognizing the C-terminal intracellular domain. The major type protein showed a band at ∼110 kd, which is consistent with the calculated molecular weight of the mature protein after the cleavage of the signal peptide, 110.8 kd (Fig. 1B). In contrast, the insCGTT type was only detected with an N-terminus antibody, but not with a C-terminus antibody, at ∼50 kd, consistent with a calculated molecular weight of 48.6 kd. We consistently found that insCGTT type expressed better likely because it is shorter and is a soluble protein (see also Fig. 1C, E, and F). We observed smaller bands, which may represent partially degraded or non-glycosylated immature forms (Fig. 1B, C, F). But because they were not always present (see Fig. 1E, 2D), we did not pursue it further.

The resultant insCGTT type protein has a signal peptide followed by an AT-D but lacks the rest of the protein (Fig. 1A). The function of this amino-terminus domain has not been fully elucidated but it is known to be involved in oligomer formation of the receptor as well as cellular trafficking, possibly through interaction with extracellular matrix proteins. We speculated that the insCGTT type protein detected in the cell homogenate might be located in the lumen of intracellular organelles found along the secretory pathway, such as the endoplasmic reticulum (ER) or Golgi apparatus. Because the N-terminus antibody of NR3B did not work well for immunostaining, we made GFP fusion proteins of NR3B major type and insCGTT type, where GFP is inserted right after the signal peptide cleavage site [31]. Hence, the resulting GFP remains attached to the protein even after signal peptide cleavage, as confirmed by western blot that showed the expected increase in molecular weight by the size of GFP (Fig. 1C). When GFP-NR3B major type or insCGTT type were expressed alone, the subcellular distribution overlapped with anti-calreticulin (CRT) immunostaining signal, a resident ER luminal protein. Additionally, there was no significant difference in the distribution of insCGTT type compared with that of major type (Fig. 1D). Co-expression of NR1 (NR1-3a splice variant) with NR3B major type or insCGTT type did not largely change the distribution (Fig. 1D). Nevertheless, co-immunoprecipitation experiments suggested that insCGTT type can interact with NR1 (Fig. 1E). Since insCGTT type has an intact AT-D domain (Fig. 1A), this result suggests that AT-D of NR3B is sufficient to associate with NR1 in a similar manner to NR1/NR2A interaction [47].

To further analyze the distribution of the insCGTT type, we treated cells with sodium carbonate solution at pH 11.5, a process which extracts luminal proteins from intracellular organelles [48]. Under this condition, CRT, a luminal protein, was extracted in the soluble fraction (lanes marked S, Fig. 1F); whereas calnexin (CNX), an integral ER membrane protein, was still associated with the membrane fraction (P), indicating that our manipulation specifically extracted luminal proteins but not membrane integral proteins. With the same treatment, insCGTT type was not extracted and was still associated with the membrane, as was the major type protein both in the presence or absence of NR1 and NR2A, suggestive of the presence of a mechanism to retain NR3B protein at the cell membrane [33].

### NR3B insCGTT type is retained on the cell surface

In HEK293T cells expressing either NR3B major type or insCGTT type, most of the signal colocalized with anti-CRT immunostaining signal, indicating that the majority of NR3B, both major type and insCGTT type, is retained intracellularly. To detect the cell surface delivery of NR3B major type and insCGTT type, we performed surface immunostaining using anti-GFP antibody under non-permeabilized conditions in live cells. To confirm if this condition allows specific detection of intracellular and extracellular pools of the protein, we first carried out immunostaining of the cells expressing GluR1 with GFP at the extracellular N-terminus and intracellular C-terminus [31]. As predicted from the membrane topology of glutamate receptors, the staining under non-permeabilized conditions showed clear surface staining in cells expressing N-terminally GFP tagged GluR1 but not in those expressing C-terminally GFP tagged GluR1 (Fig. 2A), confirming the specificity of cell surface staining.

Surface staining of the cells expressing GFP-NR3B major type with anti-GFP antibody detected an irregular spotty expression pattern (Fig. 2B). This signal did not colocalize with the majority of GFP signal, indicating that the NR3B is mostly retained intracellularly and only a small proportion is delivered to the surface. This is consistent with the observation that the GFP signal was distributed almost similarly to CRT (Fig. 1D). When NR1 was coexpressed with NR3B, we saw a slight increase in cell surface NR3B, consistent with previous reports [33,34], though it did not reach statistical significance in our study (Fig. 2B, C).

The distribution pattern of insCGTT type, both total protein detected by GFP and the cell surface protein detected by an anti-GFP antibody under non-permeabilized conditions, was similar to that of major type protein (Fig. 2B). Even though insCGTT type does not have any transmembrane domain, the surface levels of insCGTT type was ∼55% of the full-length protein (Fig. 2C). Consistently, surface expression of both NR3B major type and insCGTT type was detected using a surface biotinylation assay (Fig. 2D). α-Tubulin, an intracellular protein, was not biotinylated, confirming the specificity of surface biotinylation. Together, these data suggest that the insCGTT type still associates with the cell membrane after it is secreted into the extracellular space. It is to be determined whether the reduced surface fraction is due to reduced export via the secretary pathway or dissociation from the cell surface.

### Electrophysiological characterization of NR3B insCGTT type protein

To assess the functional impact of the insCGTT type, we investigated the electrophysiological properties of the NMDA receptor complex by co-transfecting HEK293T cells with vectors expressing major type or insCGTT type human NR3B in combination with those expressing human NR1 and NR2A subunits (Fig. 3). The current response to 1 mM glutamate (puff application, in the presence of 10 μM glycine and absence of Mg^2+^) was monitored while cells were clamped at -60 mV. The cells co-expressing NR3B with NR1 and NR2A showed a significantly smaller glutamate-evoked current than those expressing NR1 and NR2A without NR3B. In contrast, the suppression was significantly less in cells expressing the NR3B insCGTT type. Overall, these results are consistent with the previous finding obtained with mouse NR3B and confirm that the insCGTT type in human NR3B indeed leads to a functionally null [12,27,33].

### A genetic association between the NR3B insCGTT variant and schizophrenia

We next investigated an association between the insCGTT variant and schizophrenia in 586 patients with schizophrenia and 754 healthy subjects. We found significant differences in allele and genotype frequencies between the patients and control subjects (allele: p = 0.035, genotype: p = 0.045, Table 1). The minor insCGTT allele frequency was significantly higher in patients with schizophrenia (8.4%) than in controls (6.2%). When the three genotypes were divided into two genotype groups (homozygous major no insCGTT allele carrier versus carrier of one or two copies of the minor insCGTT allele), frequency of the insCGTT allele carrier was significantly higher in patients with schizophrenia than in controls (p = 0.024).

**Table 1:** Genotype and allele distributions for NR3B insCGTT type between patients with schizophrenia and controls.

Marker				SCZ (*n* = 586)			CON (*n* = 754)			Genotypic	SCZ	CON	Allelic	OR
SNP IDs	Chr Position^a^	M/m^b^	gene	M/M	M/m	m/m	M/M	M/m	m/m	*p* value	MAF		*p* value	(95% CI)
rs10666583	1004896:1004897	- / CGTT	Exon3	0.84	0.15	0.0068	0.88	0.11	0.0080	**0.045**	0.084	0.062	**0.035**	1.37(1.02–1.84)

SCZ, patients with schizophrenia; CON, healthy controls; M, major allele; m, minor allele; MAF, minor allele frequency; OR, odds ratio.

^a^db SNP build 129.

^b^The first alleles shown are major alleles. The allele is represented according to the plus strand DNA sequence.

Significant *p* values are shown as bold-faced and underlined type.

### Impact of the insCGTT variant on three phenotypes related to schizophrenia

Based on genetic association analysis, we examined replication analyses of associations between the insCGTT variant (insCGTT carriers versus individuals without insCGTT) and three phenotypes related to schizophrenia in 121 patients with schizophrenia and 372 healthy subjects. In demographic variables, mean age did not differ significantly between the patients and controls (*p* = 0.51), while the female ratio, years of education and estimated premorbid intelligence quotient were significantly lower in the patients with schizophrenia compared to controls (*p*<0.002) (Table 2).

**Table 2:** Demographic variables for subjects included in the SPQ, PPI and WCST analyses.

	Schizophrenia	Controls		*p* values	(*z*)
Variable	(*n* = 121)	(*n* = 372)			
Age (years)	36.7 ± 1.2	35.9 ± 0.6		0.51	0.65
Gender (male/female)^a^	75/46	170/202		**0.002**	9.69
Education (years)	13.8 ± 0.2	15.0 ± 0.1		**<0.001**	-4.48
Estimated premorbid IQ	101.7 ± 1.0	107.3 ± 0.4		**<0.001**	-5.19
CPZeq. (mg/day)	599.2 ± 48.0	-		-	-
Age at onset (years)	23.7 ± 0.8	-		-	-
Duration of illness (years)	13.0 ± 1.0	-		-	-
PANSS positive symptoms	19.1 ± 0.5	-		-	-
PANSS negative symptoms	20.0 ± 0.6	-		-	-
PANSS general psychopathology	40.8 ± 1.0	-		-	-

IQ: intelligence quotient; CPZeq: chlorpromazine equivalents; PANSS: Positive and Negative Syndrome Scale.

Means ± SE are shown. *P* values < 0.05 are in boldface and underlined.

^a^ *χ* ^*2*^ test.

**Fig 4:**
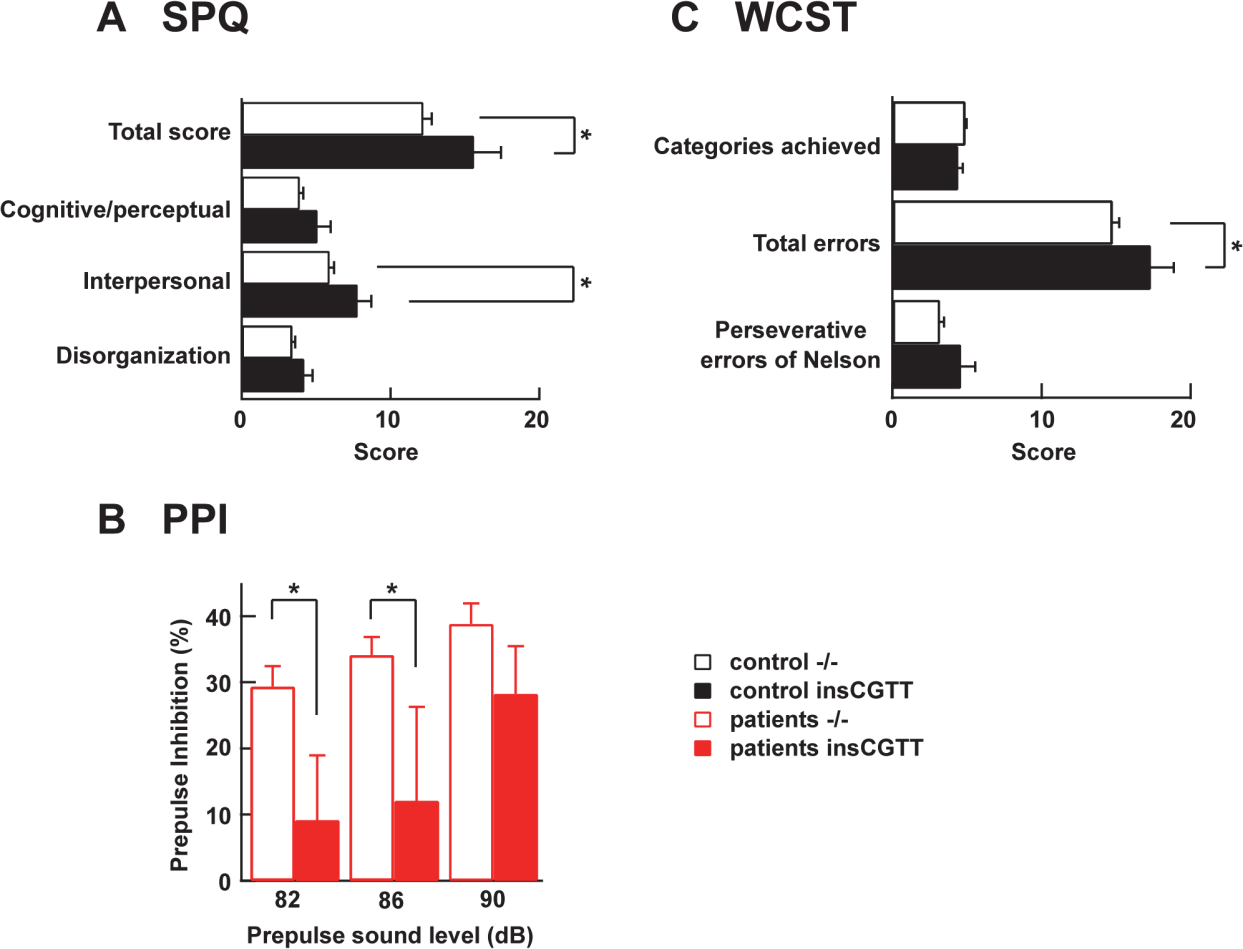
Genetic analyses of insCGTT type in patients with schizophrenia and controls. **A**, The association between the NR3B insCGTT type and SPQ total score and the three factors in control individuals. -/-, n = 317; insCGTT carrier, n = 44. **B**, The association between NR3B insCGTT type and PPI in schizophrenia patients. -/-, n = 107; insCGTT carrier, n = 14 in patients with schizophrenia. **C**, The association between NR3B insCGTT type and WCST score in control individuals. -/-, n = 243; insCGTT carrier, n = 31. *: p<0.05.

First, we investigated the impact of the variant on schizotypal personality traits in 361 healthy subjects using the Schizotypal Personality Questionnaire (SPQ). We found a significant effect of the insCGTT variant on the total SPQ score (*F*_*1*,*356*_ = 4.04, *p* = 0.045, Fig. 4A). We then investigated the genotype effects on the three SPQ factors, cognitive/perceptual, interpersonal and disorganization. A significant genotype effect was observed on the interpersonal factor (*F*_*1*,*356*_ = 4.69, *p* = 0.031. Fig. 4A), but no significant genotype effects were observed on the cognitive/perceptual or disorganization factors (*p*>0.10). Individuals with the risk-associated insCGTT genotype showed higher scores on schizotypal traits, particularly the interpersonal factor, compared with those without insCGTT.

Second, we examined if there was any association between the variant and PPI in 121 patients with schizophrenia. There was no difference in acoustic startle reflex or habituation between genotype groups (*p*>0.20, not shown). However, there were significant effects of the variant on two pre-pulse intensities, PPI82 and PPI86 (PPI82: *F*_*1*,*116*_ = 4.51; *p* = 0.036, PPI86: *F*_*1*,*116*_ = 5.72; *p* = 0.018, PPI90: *F*_*1*,*116*_ = 0.99; *p* = 0.32). Patients with insCGTT carriers showed significant deficits in PPI than those without insCGTT (Fig. 4B).

Third, we investigated the impact of the variant on executive function measured by the Wisconsin Card Sorting Test (WCST) in 243 healthy subjects (Fig. 4C). We found a significant effect of insCGTT variant on the number of total errors (*F*_*1*,*238*_ = 4.46, *p* = 0.036) and marginal effects of the insCGTT variant on the number of categories achieved (*F*_*1*,*238*_ = 3.66, *p* = 0.057) and the perseverative errors of Nelson type (*F*_*1*,*238*_ = 3.69, *p* = 0.056). Individuals with the insCGTT variant showed impaired executive functions compared with those without the insCGTT allele.

Overall, the results of the genetic analysis of the insCGTT allele in Japanese schizophrenia patients and healthy individuals indicate that the functional loss of NR3B by insCGTT might be a risk factor of schizophrenia symptoms. Our attempt of three replication analyses of schizophrenia related phenotypes supported the association between insCGTT allele and schizophrenia.

## Discussion

In this study, using genetic approaches, we assessed if a naturally occurring null mutant of NR3B, one of the modulatory subunits of NMDAR, has any impact on the pathogenesis of schizophrenia in the Japanese population. The variant causes a frame-shift, resulting in a truncated protein that contains the full AT-D, with homology to the bacterial periplasmic binding protein, but not the rest of the protein (Fig. 1A). In glutamate receptor, instead of binding to a ligand, this domain appears to be involved in protein interaction and allosterically modulates channel open probability and determines the subcellular localization [49].

When the NR3B insCGTT type was expressed in heterologous cells, much of the protein was trapped in intracellular organelles, likely at the endoplasmitic reticulum (Fig. 1D) or Golgi apparatus in a form that cannot be readily extracted under alkaline conditions that normally extract luminal proteins (Fig. 1F). Also the NR3B insCGTT type associates with the cell surface, to approximately 50% of the major type protein (Fig. 2C, D). Given that this variation does not have a transmembrane or membrane associated domain, the remaining extracellular domain in the NR3B insCGTT type most likely accounts for this property. Co-immunoprecipitation data demonstrated that NR3B insCGTT type can interact with the NR1 subunit (Fig. 1E) and suggested that NR1 may associate with NR3B insCGTT on the ER membrane, and a part of the complex can leave the ER then stably localize in the cell membrane. Alternatively, a binding protein functionally similar to pentraxin for AMPA type glutamate receptors [9,50] may exist for NR3B and limit the dissociation from the cellular membrane. However, this remaining portion of the protein is apparently non-functional as demonstrated with electrophysiological recordings. It remains to be shown whether such translation products exist or not in the neuronal tissue of carriers. It is also possible that the insCGTT type mRNA is subject to non-sense-mediated mRNA decay [51]. In either case, *in vivo*, the NR3B insCGTT type results in a functionally null allele in carriers.

Among schizophrenia patients and healthy controls, the NR3B insCGTT type was significantly overrepresented in the patients compared with controls. Also, NR3B insCGTT type was associated with impaired performance in schizophrenia related phenotypes—SPQ, PPI and WCST. We previously found that the *Grin3b* knockout mouse have a phenotype suggestive of increased anxiety, such as decreased entry and time spent in the open arm of the elevated plus maze task [28]. The animals showed an overall tendency for a reduction in pre-pulse inhibition compared with wild type animals, which is consistent with our finding in schizophrenia patients, though this effect did not reach statistical significance [28]. However, the mice showed behavior suggestive of increased social interaction in the home cage. While this indicates that NR3B plays a role in the regulation of social behavior, it is not completely consistent with the schizophrenia phenotype seen in humans. This may be due to species differences in the function or expression pattern of NR3B. Indeed, *GRIN3B* is one of the neuronal genes which has an accelerated evolution in primate lineage (including human) compared with rodent lineage [27]. The homology between human and mouse NR3B is 79.1% at the amino acid level, while that of other subunits of ionotrophic glutamate receptors is higher. For example, NR1 has 99.0%, NR2A has 95.3%, and NR3A has 93.4% homology between these two species. Therefore, it is possible that NR3B has specific roles in humans, which cannot be reproduced in the animal model. The functional loss of NR3B will lead to an increase in the glutamatergic current mediated by NR1/NR2/NR3 channels or a decrease in the glycinergic current mediated by NR1/NR3B synapses. The contribution of these two mechanisms to the pathogenesis of schizophrenia is yet to be determined. Also, the brain region and cell types that are affected by the loss of NR3B function and critical for the pathogenesis of schizophrenia are still unclear. Whereas abundant expression is restricted to α-motoneurons in the brainstem and spinal cord [12,13,23], there is diffuse distribution in multiple brain regions [22,26,52]. Future studies are required to fully address these questions.

The InsCGTT variant has not been reported as a risk mutation in past genome wide association studies (GWAS). This could be false negative results or a real risk mutation of very small effect. Polygenic risk score analysis revealed that many risk variations with very small effect may not be detected by GWAS with a very strict p value threshold (p < 5 x 10^–8^) [53]. As our sample size is limited and our results are derived from a Japanese sample, it is important to conduct further investigations to confirm these findings in other larger samples and/or with different ethnicities. Indeed, in contrast to Japanese and other East Asian populations, the incidence of insCGTT is much higher in the European descendants [27]. In a mixed European descendant population in the United States of America, among 362 apparently healthy individuals, we found 138 insCGTT/- carriers (38.1%) and 38 insCGTT/insCGTT carriers (10.5%), which gives an allele frequency of insCGTT 0.296 [27]. It has occasionally been reported that the differences in risk-allele frequencies, such as genetic variation in the *TCF4* gene and MHC region, for schizophrenia between East Asian and European populations [53]. These differences might mean that there are some genetic variants that contribute to the susceptibility for schizophrenia in a different manner among ethnicities.

Several molecular genetic studies have investigated the influences of susceptibility genes for schizophrenia on schizotypal personality traits. These studies have reported associations between the *COMT* [54–56], *DTNBP1* [57,58], *DAO* [58], *ZNF804A* [59] and *ARHGAP32* (also known as p250GAP) [59] genes and schizotypal components. Risk alleles or haplotypes of schizophrenia were correlated with high scores on schizotypal personality traits. Of these genes, the *DTNBP1*, *DAO*, *and ARHGAP32* genes, in addition to the different NMDA receptor subunit genes [15–20,21]; and this study] are directly responsible for mediating NMDAR signaling. However, involvement of the glutamate NMDARs in SPQ is still unknown. Further research will need to clarify the relationship between the NMDAR and SPQ. Several studies reported the association between PPI in schizophrenia and *HT2AR*, *COMT*, *NRG1* and *RELA* [60–62], which also directly or indirectly influence glutamatergic signaling.

WCST has been well established as a test for working memory reflecting prefrontal information processing. The performance of WCST shows good association with Val158Met polymorphism of *COMT* [63], an enzyme involved in dopamine metabolism. Our data suggests an association between WCST performance and NMDAR signaling, which is consistent with a previous study showing an association between WCST performance and *MAGI2* (also known as S-SCAM), a scaffolding protein at glutamatergic synapses [45].

In summary, we found that genetic loss of NR3B itself is not pathogenic but constitutes a risk factor of schizophrenia in the Japanese population. Likely in combination with other genetic or environmental factors, this loss of NR3B can potentially lead to the onset of schizophrenia.

### Note added in proof

After the revised manuscript was submitted to re-review, a publication by Lin et al. came to our attention [64]. In this work, the authors found a SNP RS2240158 of GRIN3B was significantly associated with mismatch negativity, a proposed endophenotype of schizophrenia, in healthy subjects. RS2240158 encodes a missense mutation c.1730C>T (T577M), which may affect protein function. However, because both insCGTT and RS2240158 are on the same exon 3 of GRIN3B [65], it is also possible the effect Lin et al. observed was due to insCGTT, rather than RS2240158 itself.

## Supporting Information

S1 Table SNPs found in NR3B cDNA used in this study. (PDF)

## References

[1] Pulay AJ, Stinson FS, Dawson DA, Goldstein RB, Chou SP, et al (2009) Prevalence, correlates, disability, and comorbidity of DSM-IV schizotypal personality disorder: results from the wave 2 national epidemiologic survey on alcohol and related conditions. Prim Care Companion J Clin Psychiatry 11: 53–67. 19617934 10.4088/pcc.08m00679 PMC2707116

[2] Snyder MA, Gao WJ (2013) NMDA hypofunction as a convergence point for progression and symptoms of schizophrenia. Front Cell Neurosci 7: 31 10.3389/fncel.2013.00031 23543703 PMC3608949

[3] Anis NA, Berry SC, Burton NR, Lodge D (1983) The dissociative anaesthetics, ketamine and phencyclidine, selectively reduce excitation of central mammalian neurones by N-methyl-aspartate. Br J Pharmacol 79: 565–575. 6317114 10.1111/j.1476-5381.1983.tb11031.x PMC2044888

[4] Javitt DC, Zukin SR (1991) Recent advances in the phencyclidine model of schizophrenia. Am J Psychiatry 148: 1301–1308. 1654746 10.1176/ajp.148.10.1301

[5] Mohn AR, Gainetdinov RR, Caron MG, Koller BH (1999) Mice with reduced NMDA receptor expression display behaviors related to schizophrenia. Cell 98: 427–436. 10.1016/S0092-8674(00)81972-8 10481908

[6] Belforte JE, Zsiros V, Sklar ER, Jiang Z, Yu G, et al (2010) Postnatal NMDA receptor ablation in corticolimbic interneurons confers schizophrenia-like phenotypes. Nat Neurosci 13: 76–83. 10.1038/nn.2447 19915563 PMC2797836

[7] Akbarian S, Sucher NJ, Bradley D, Tafazzoli A, Trinh D, et al (1996) Selective alterations in gene expression for NMDA receptor subunits in prefrontal cortex of schizophrenics. J Neurosci 16: 19–30. 8613785 10.1523/JNEUROSCI.16-01-00019.1996 PMC6578738

[8] Cioffi CL (2013) Modulation of NMDA receptor function as a treatment for schizophrenia. Bioorg Med Chem Lett 23: 5034–5044. 10.1016/j.bmcl.2013.07.019 23916256

[9] Traynelis SF, Wollmuth LP, McBain CJ, Menniti FS, Vance KM, et al (2010) Glutamate receptor ion channels: structure, regulation, and function. Pharmacol Rev 62: 405–496. 10.1124/pr.109.002451 20716669 PMC2964903

[10] Ciabarra AM, Sullivan JM, Gahn LG, Pecht G, Heinemann S, et al (1995) Cloning and characterization of chi-1: a developmentally regulated member of a novel class of the ionotropic glutamate receptor family. J Neurosci 15: 6498–6508. 7472412 10.1523/JNEUROSCI.15-10-06498.1995 PMC6577996

[11] Sucher NJ, Akbarian S, Chi CL, Leclerc CL, Awobuluyi M, et al (1995) Developmental and regional expression pattern of a novel NMDA receptor- like subunit (NMDAR-L) in the rodent brain. J Neurosci 15: 6509–6520. 7472413 10.1523/JNEUROSCI.15-10-06509.1995 PMC6578025

[12] Nishi M, Hinds H, Lu HP, Kawata M, Hayashi Y (2001) Motoneuron-specific expression of NR3B, a novel NMDA-type glutamate receptor subunit that works in a dominant-negative manner. J Neurosci 21: RC185 11717388 10.1523/JNEUROSCI.21-23-j0003.2001 PMC6763906

[13] Chatterton JE, Awobuluyi M, Premkumar LS, Takahashi H, Talantova M, et al (2002) Excitatory glycine receptors containing the NR3 family of NMDA receptor subunits. Nature 415: 793–798. 10.1038/nature715 11823786

[14] Riley B, Kendler KS (2006) Molecular genetic studies of schizophrenia. Eur J Hum Genet 14: 669–680. 10.1038/sj.ejhg.5201571 16721403

[15] Begni S, Moraschi S, Bignotti S, Fumagalli F, Rillosi L, et al (2003) Association between the G1001C polymorphism in the GRIN1 gene promoter region and schizophrenia. Biol Psychiatry 53: 617–619. 10.1016/S0006-3223(02)01783-3 12679240

[16] Galehdari H, Pooryasin A, Foroughmand A, Daneshmand S, Saadat M (2009) Association between the G1001C polymorphism in the GRIN1 gene promoter and schizophrenia in the Iranian population. J Mol Neurosci 38: 178–181. 10.1007/s12031-008-9148-5 18792810

[17] Martucci L, Wong AH, De Luca V, Likhodi O, Wong GW, et al (2006) N-methyl-D-aspartate receptor NR2B subunit gene GRIN2B in schizophrenia and bipolar disorder: Polymorphisms and mRNA levels. Schizophr Res 84: 214–221. 10.1016/j.schres.2006.02.001 16549338

[18] Qin S, Zhao X, Pan Y, Liu J, Feng G, et al (2005) An association study of the N-methyl-D-aspartate receptor NR1 subunit gene (GRIN1) and NR2B subunit gene (GRIN2B) in schizophrenia with universal DNA microarray. Eur J Hum Genet 13: 807–814. 10.1038/sj.ejhg.5201418 15841096

[19] Allen NC, Bagade S, McQueen MB, Ioannidis JP, Kavvoura FK, et al (2008) Systematic meta-analyses and field synopsis of genetic association studies in schizophrenia: the SzGene database. Nat Genet 40: 827–834. 10.1038/ng.171 18583979

[20] Tarabeux J, Kebir O, Gauthier J, Hamdan FF, Xiong L, et al (2011) Rare mutations in N-methyl-D-aspartate glutamate receptors in autism spectrum disorders and schizophrenia. Transl Psychiatry 1: e55 10.1038/tp.2011.52 22833210 PMC3309470

[21] Takata A, Iwayama Y, Fukuo Y, Ikeda M, Okochi T, et al (2013) A population-specific uncommon variant in GRIN3A associated with schizophrenia. Biol Psychiatry 73: 532–539. 10.1016/j.biopsych.2012.10.024 23237318

[22] Matsuda K, Kamiya Y, Matsuda S, Yuzaki M (2002) Cloning and characterization of a novel NMDA receptor subunit NR3B: a dominant subunit that reduces calcium permeability. Brain Res Mol Brain Res 100: 43–52. 12008020 10.1016/s0169-328x(02)00173-0

[23] Fukaya M, Hayashi Y, Watanabe M (2005) NR2 to NR3B subunit switchover of NMDA receptors in early postnatal motoneurons. Eur J Neurosci 21: 1432–1436. 10.1111/j.1460-9568.2005.03957.x 15813953

[24] Low CM, Wee KS (2010) New insights into the not-so-new NR3 subunits of N-methyl-D-aspartate receptor: localization, structure, and function. Mol Pharmacol 78: 1–11. 10.1124/mol.110.064006 20363861

[25] Andersson O, Stenqvist A, Attersand A, von Euler G (2001) Nucleotide sequence, genomic organization, and chromosomal localization of genes encoding the human NMDA receptor subunits NR3A and NR3B. Genomics 78: 178–184. 10.1006/geno.2001.6666 11735224

[26] Bendel O, Meijer B, Hurd Y, von Euler G (2005) Cloning and expression of the human NMDA receptor subunit NR3B in the adult human hippocampus. Neurosci Lett 377: 31–36. 10.1016/j.neulet.2004.11.064 15722182

[27] Niemann S, Landers JE, Churchill MJ, Hosler B, Sapp P, et al (2008) Motoneuron-specific NR3B gene: no association with ALS and evidence for a common null allele. Neurology 70: 666–676. 10.1212/01.wnl.0000271078.51280.17 17687115

[28] Niemann S, Kanki H, Fukui Y, Takao K, Fukaya M, et al (2007) Genetic ablation of NMDA receptor subunit NR3B in mouse reveals motoneuronal and nonmotoneuronal phenotypes. Eur J Neurosci 26: 1407–1420. 10.1111/j.1460-9568.2007.05774.x 17880385

[29] Smothers CT, Woodward JJ (2009) Expression of glycine-activated diheteromeric NR1/NR3 receptors in human embryonic kidney 293 cells Is NR1 splice variant-dependent. J Pharmacol Exp Ther 331: 975–984. 10.1124/jpet.109.158493 19726695 PMC2784711

[30] Fukumori R, Takarada T, Nakamichi N, Kambe Y, Kawagoe H, et al (2010) Requirement of both NR3A and NR3B subunits for dominant negative properties on Ca2+ mobilization mediated by acquired N-methyl-D-aspartate receptor channels into mitochondria. Neurochem Int 57: 730–737. 10.1016/j.neuint.2010.08.009 20813147

[31] Shi SH, Hayashi Y, Petralia RS, Zaman SH, Wenthold RJ, et al (1999) Rapid spine delivery and redistribution of AMPA receptors after synaptic NMDA receptor activation. Science 284: 1811–1816. 10.1126/science.284.5421.1811 10364548

[32] Niwa H, Yamamura K, Miyazaki J (1991) Efficient selection for high-expression transfectants with a novel eukaryotic vector. Gene 108: 193–199. 1660837 10.1016/0378-1119(91)90434-d

[33] Matsuda K, Fletcher M, Kamiya Y, Yuzaki M (2003) Specific assembly with the NMDA receptor 3B subunit controls surface expression and calcium permeability of NMDA receptors. J Neurosci 23: 10064–10073. 14602821 10.1523/JNEUROSCI.23-31-10064.2003 PMC6740865

[34] Wee KS, Wee ZN, Chow NB, Low CM (2010) The distal carboxyl terminal of rat NR3B subunit regulates NR1-1a/NR3B and NR1-2a/NR3B surface trafficking. Neurochem Int 57: 97–101. 10.1016/j.neuint.2010.05.003 20466026

[35] Hashimoto R, Hashimoto H, Shintani N, Chiba S, Hattori S, et al (2007) Pituitary adenylate cyclase-activating polypeptide is associated with schizophrenia. Molecular Psychiatry 12: 1026–1032. 10.1038/sj.mp.4001982 17387318

[36] Hashimoto R, Numakawa T, Ohnishi T, Kumamaru E, Yagasaki Y, et al (2006) Impact of the DISC1 Ser704Cys polymorphism on risk for major depression, brain morphology and ERK signaling. Human Molecular Genetics 15: 3024–3033. 10.1093/hmg/ddl244 16959794

[37] Iijima Y, Sasaki J, Bando N, Asai T, Mouri I, et al (2010) Development of a Japanese version of the schizotypal personality questionnaire and factor structure of schizotypy. Koudouryouhoukenkyu 36: 29–41.

[38] Someya T, Sasaki T, Takahashi S (1994) Reliability and validity of schizotypal personality questionnaire (in Japanese). The Proceeding of the 32nd Scientific Meeting of the University Health Care in Japan 286–290.

[39] Raine A (1991) The SPQ: a scale for the assessment of schizotypal personality based on DSM-III-R criteria. Schizophr Bull 17: 555–564. 1805349 10.1093/schbul/17.4.555

[40] Raine A, Reynolds C, Lencz T, Scerbo A, Triphon N, et al (1994) Cognitive-perceptual, interpersonal, and disorganized features of schizotypal personality. Schizophr Bull 20: 191–201. 8197415 10.1093/schbul/20.1.191

[41] Moriwaki M, Kishi T, Takahashi H, Hashimoto R, Kawashima K, et al (2009) Prepulse inhibition of the startle response with chronic schizophrenia: a replication study. Neurosci Res 65: 259–262. 10.1016/j.neures.2009.07.009 19660506

[42] Takahashi H, Iwase M, Canuet L, Yasuda Y, Ohi K, et al (2010) Relationship between prepulse inhibition of acoustic startle response and schizotypy in healthy Japanese subjects. Psychophysiology 47: 831–837. 10.1111/j.1469-8986.2010.01000.x 20233344

[43] Takahashi H, Iwase M, Ishii R, Ohi K, Fukumoto M, et al (2008) Impaired prepulse inhibition and habituation of acoustic startle response in Japanese patients with schizophrenia. Neurosci Res 62: 187–194. 10.1016/j.neures.2008.08.006 18789980

[44] Hashimoto R, Ohi K, Yasuda Y, Fukumoto M, Yamamori H, et al (2011) Variants of the RELA gene are associated with schizophrenia and their startle responses. Neuropsychopharmacology 36: 1921–1931. 10.1038/npp.2011.78 21593732 PMC3154111

[45] Koide T, Banno M, Aleksic B, Yamashita S, Kikuchi T, et al (2012) Common variants in MAGI2 gene are associated with increased risk for cognitive impairment in schizophrenic patients. PLoS One 7: e36836 10.1371/journal.pone.0036836 22649501 PMC3359314

[46] Banno M, Koide T, Aleksic B, Yamada K, Kikuchi T, et al (2011) A case control association study and cognitive function analysis of neuropilin and tolloid-like 1 gene and schizophrenia in the Japanese population. PLoS One 6: e28929 10.1371/journal.pone.0028929 22205981 PMC3243668

[47] Meddows E, Le Bourdelles B, Grimwood S, Wafford K, Sandhu S, et al (2001) Identification of molecular determinants that are important in the assembly of N-methyl-D-aspartate receptors. J Biol Chem 276: 18795–18803. 10.1074/jbc.M101382200 11279200

[48] Fujiki Y, Hubbard AL, Fowler S, Lazarow PB (1982) Isolation of intracellular membranes by means of sodium carbonate treatment: application to endoplasmic reticulum. J Cell Biol 93: 97–102. 10.1083/jcb.93.1.97 7068762 PMC2112113

[49] Gielen M, Siegler Retchless B, Mony L, Johnson JW, Paoletti P (2009) Mechanism of differential control of NMDA receptor activity by NR2 subunits. Nature 459: 703–707. 10.1038/nature07993 19404260 PMC2711440

[50] O'Brien RJ, Xu D, Petralia RS, Steward O, Huganir RL, et al (1999) Synaptic clustering of AMPA receptors by the extracellular immediate-early gene product Narp. Neuron 23: 309–323. 10.1016/S0896-6273(00)80782-5 10399937

[51] Baker KE, Parker R (2004) Nonsense-mediated mRNA decay: terminating erroneous gene expression. Curr Opin Cell Biol 16: 293–299. 10.1016/j.ceb.2004.03.003 15145354

[52] Wee KS, Zhang Y, Khanna S, Low CM (2008) Immunolocalization of NMDA receptor subunit NR3B in selected structures in the rat forebrain, cerebellum, and lumbar spinal cord. J Comp Neurol 509: 118–135. 10.1002/cne.21747 18425811

[53] International Schizophrenia C, Purcell SM, Wray NR, Stone JL, Visscher PM, et al (2009) Common polygenic variation contributes to risk of schizophrenia and bipolar disorder. Nature 460: 748–752. 10.1038/nature08185 19571811 PMC3912837

[54] Avramopoulos D, Stefanis NC, Hantoumi I, Smyrnis N, Evdokimidis I, et al (2002) Higher scores of self reported schizotypy in healthy young males carrying the COMT high activity allele. Mol Psychiatry 7: 706–711. 10.1038/sj.mp.4001070 12192614

[55] Schurhoff F, Szoke A, Chevalier F, Roy I, Meary A, et al (2007) Schizotypal dimensions: an intermediate phenotype associated with the COMT high activity allele. Am J Med Genet B Neuropsychiatr Genet 144B: 64–68. 10.1002/ajmg.b.30395 17034018

[56] Sheldrick AJ, Krug A, Markov V, Leube D, Michel TM, et al (2008) Effect of COMT val158met genotype on cognition and personality. Eur Psychiatry 23: 385–389. 10.1016/j.eurpsy.2008.05.002 18755576

[57] Kircher T, Markov V, Krug A, Eggermann T, Zerres K, et al (2009) Association of the DTNBP1 genotype with cognition and personality traits in healthy subjects. Psychol Med 39: 1657–1665. 10.1017/S0033291709005388 19335929

[58] Stefanis NC, Trikalinos TA, Avramopoulos D, Smyrnis N, Evdokimidis I, et al (2007) Impact of schizophrenia candidate genes on schizotypy and cognitive endophenotypes at the population level. Biol Psychiatry 62: 784–792. 10.1016/j.biopsych.2006.11.015 17336946

[59] Yasuda Y, Hashimoto R, Ohi K, Fukumoto M, Umeda-Yano S, et al (2011) Impact on schizotypal personality trait of a genome-wide supported psychosis variant of the ZNF804A gene. Neurosci Lett 495: 216–220. 10.1016/j.neulet.2011.03.069 21457757

[60] Hong LE, Wonodi I, Stine OC, Mitchell BD, Thaker GK (2008) Evidence of missense mutations on the neuregulin 1 gene affecting function of prepulse inhibition. Biol Psychiatry 63: 17–23. 10.1016/j.biopsych.2007.05.011 17631867 PMC3569848

[61] Quednow BB, Wagner M, Mossner R, Maier W, Kuhn KU (2010) Sensorimotor gating of schizophrenia patients depends on Catechol O-methyltransferase Val158Met polymorphism. Schizophr Bull 36: 341–346. 10.1093/schbul/sbn088 18635674 PMC2833112

[62] Quednow BB, Kuhn KU, Mossner R, Schwab SG, Schuhmacher A, et al (2008) Sensorimotor gating of schizophrenia patients is influenced by 5-HT2A receptor polymorphisms. Biol Psychiatry 64: 434–437. 10.1016/j.biopsych.2008.02.019 18420180

[63] Egan MF, Goldberg TE, Kolachana BS, Callicott JH, Mazzanti CM, et al (2001) Effect of COMT Val108/158 Met genotype on frontal lobe function and risk for schizophrenia. Proc Natl Acad Sci U S A 98: 6917–6922. 10.1073/pnas.111134598 11381111 PMC34453

[64] Lin YT, Hsieh MH, Liu CC, Hwang TJ, Chien YL, et al (2014) A recently-discovered NMDA receptor gene, GRIN3B, is associated with duration mismatch negativity. Psychiatry Res 218: 356–358. 10.1016/j.psychres.2014.04.032 24814139

[65] Niemann S, Landers JE, Churchill MJ, Hosler B, Sapp P, et al (2008) Motoneuron-specific NR3B gene: no association with ALS and evidence for a common null allele. Neurology 70: 666–676. 17687115 10.1212/01.wnl.0000271078.51280.17

