# Supplementary material for: A Naturally Occurring Null Variant of the NMDA Type Glutamate Receptor NR3B Subunit Is a Risk Factor of Schizophrenia
Source: PLoS One. 2015 Mar 13;10(3):e0116319. doi: 10.1371/journal.pone.0116319 (PMC4358936; doi:10.1371/journal.pone.0116319)
Supplement: S1 Table — (PDF) [file pone.0116319.s001.pdf]

**Supporting Information**

**Table S1. SNPs found in NR3B cDNA used in this study.**

| Nucleotide change | Amino acid change | dbSNP and references |
|---|---|---|
| c.132C>A | P44P | (Niemann et al., 2008) |
| c.325G>T | A109S | unreported |
| c.349C>A | H117Y | rs12986002 (Niemann et al., 2008) |
| c.363G>C | A121A | rs2301813 (Niemann et al., 2008) |
| c.1210C>T | R404W | rs4807399 (Niemann et al., 2008) |
| c.1224A>G | P408P | rs11880849 (Niemann et al., 2008) |
| c.1240T>C | W414R | rs2240157 (Niemann et al., 2008) |
| c.1323G>A | A441A | rs4806908 (Niemann et al., 2008) |
| c.1344T>C | P448P | rs4806909 (Niemann et al., 2008) |
| c.1372G>A | A458T | unreported |
| c.1730C>T | T577M | rs2240158 (Niemann et al., 2008) |
| c.1834A>G | T612A | unreported |
| c.2896T>A | Y966N | rs10417824 (Niemann et al., 2008) |
| c.3016C>G | Q1006E | rs10401245 (Niemann et al., 2008) |
| c.3018-3108del | | (Niemann et al., 2008) |
